# Isoflavone daidzein ameliorates renal dysfunction and fibrosis in a postmenopausal rat model: Intermediation of angiotensin AT1 and Mas receptors and microRNAs 33a and 27a

**DOI:** 10.22038/IJBMS.2022.66572.14609

**Published:** 2022-11

**Authors:** Majid Askaripour, Hamid Najafipour, Shadan Saberi, Saleh Yazdani, Saeideh Jafarinejad-Farsangi, Soodeh Rajabi, Elham Jafari, Paul Proost, Sofie Struyf, Fariba Poosti

**Affiliations:** 1 Department of Physiology and Pharmacology, and Cardiovascular Research Center, Institute of Basic and Clinical Physiology Sciences, Kerman University of Medical Sciences, Kerman, Iran; 2 Department of Physiology and Pharmacology, Afzalipour Medical Faculty and Physiology Research Centre, Kerman University of Medical Sciences, Kerman, Iran; 3 VIB-KU Leuven Center for Microbiology, Leuven, Belgium; 4 Laboratory of Molecular Cell Biology, Department of Biology, KU Leuven, Leuven, Belgium; 5 Physiology Research Centre, Institute of Neuropharmacology, Kerman University of Medical Sciences, Kerman, Iran; 6 Endocrinology and Metabolism Research Center, Institute of Basic and Clinical Physiology Sciences, Kerman University of Medical Sciences, Kerman, Iran; 7 Pathology and Stem Cell Research Center, Department of Pathology, Kerman University of Medical Sciences, Kerman, Iran; 8 Laboratory of Molecular Immunology, Department of Microbiology, Immunology, and Transplantation, Rega Institute for Medical Research, KU Leuven, Leuven, Belgium

**Keywords:** Angiotensin receptor, Apoptosis, Daidzein, microRNAs, Ovariectomy, Renal fibrosis

## Abstract

**Objective(s):**

Chronic kidney disease (CKD), accompanied by renal dysfunction, fibrosis, and apoptosis, is highly prevalent in postmenopausal women. We tested the hypothesis that isoflavone daidzein may ameliorate renal dysfunction and fibrosis through angiotensin II type 1 (AT1R) and angiotensin 1-7 (MasR) receptors in association with microRNAs 33a and 27a.

**Materials and Methods:**

Two weeks before the initiation of the experiments, rats (n=84) underwent ovariectomy (OVX). Then, unilateral ureteral obstruction (UUO) was performed in OVX rats, and animals were allocated to the following groups (n=21): sham vehicle (dimethyl sulfoxide; DMSO 1%), UUO vehicle, UUO+17β-estradiol (E2), and UUO+daidzein. Each group encompassed three subgroups (n=7) treated with saline, A779 (MasR antagonist), or losartan (AT1R antagonist) for 15 days. The fractional urine excretion of sodium (FE_Na+_) and potassium (FE_K+_), renal failure index (RFI), renal interstitial fibrosis (RIF index), glomerulosclerosis, miR-33a, and miR-27a expressions and their target genes were analyzed. Apoptosis was measured via cleaved caspase-3 immunohistochemistry.

**Results:**

UUO increased kidney weight, FE_Na+_, FE_K+_, urine calcium, RFI, RIF index, glomerulosclerosis, and cleaved caspase-3. Moreover, expression of renal miR-33a and miR-27a, collagen3A1 mRNA, and protein were up-regulated post-UUO. Daidzein treatment alleviated the harmful effects of UUO especially in co-treatment with losartan. They also masked the anticipated worsening effects of A779 on UUO.

**Conclusion:**

Compared with E2, daidzein efficiently ameliorated renal dysfunction, fibrosis, and apoptosis through modulation of miR-33a and miR-27a expression and their crosstalk with AT1R and MasR. Therefore, daidzein might be a promising candidate for treating CKD in postmenopausal and older women.

## Introduction

Renal fibrosis is the primary pathological process of CKD, which is characterized by excessive deposition of extracellular matrix (ECM) (1). Renal fibrosis is accompanied by a loss of renal epithelial cells due to up-regulation of angiotensin II (Ang II) type I receptor (AT1R) expression, down-regulation of angiotensin 1-7 (Ang1-7)/MasR, activation of transforming growth factor-β1 (TGF-β1), microRNA (miR) under- or overexpression, inflammation, epithelial-to-mesenchymal transition, and fibroblast to myofibroblast trans-differentiation (2). The experimental unilateral ureteral obstruction (UUO) model of obstructive nephropathy has been widely used to investigate the mechanism of renal fibrosis (3).

miRNAs are non-coding RNAs that have gained attention in the pathophysiology of UUO-induced renal fibrosis (4). However, their precise role in molecular mechanisms of renal fibrosis is still not fully elucidated (5). The pro-fibrotic miR-33a and miR-27a play an influential role in metabolic disorders, apoptosis, and fibrosis (5, 6).

The renin-angiotensin system (RAS) is a crucial factor in the development and progression of renal fibrosis and tubular apoptosis in obstructive nephropathy (7). Ang II is critically involved in the pathogenesis of renal fibrosis and apoptosis via cytokine activation, inflammation, and myofibroblast trans-differentiation (7). Despite the harmful effects of AngII, the non-classical Ang1–7/Mas receptor axis, provides protection against renal dysfunction, fibrosis, apoptosis, and glomerulosclerosis, and the MasR antagonist (A779) or gene deletion of the MasR inhibited these beneficial effects (8, 9). The expression of RAS pathway components is gender-dependent and higher in men than women, while the Ang1–7/MasR axis expression is contrariwise (10). E2 plays a role in this difference since after menopause the risk of CKD in women equals that of men and is even more prominent (1). Hence, one of the effective treatment strategies in postmenopausal women is estrogen-based hormone replacement therapy (HRT) (11). Some studies have shown the protective role of 17β-estradiol (E2) in fibrotic diseases by modulating the RAS axis (12). However, several side effects of HRT in postmenopausal women over 65 years have been reported, including heart diseases, breast cancer, endometrial cancer, and pulmonary embolism (13). Therefore, the replacement of HRT with natural molecules having the same beneficial effects as E2 with fewer/no side effects would be a new strategy for CKD treatment (14).

Phytoestrogens are polyphenolic plant-based substances with a similar structure to E2 and the capability to bind E2 receptors (15). Clinical and experimental studies have shown that phytoestrogens reduce the risk of cardiovascular disease, CKD, diabetes mellitus, osteoporosis, menopausal symptoms, breast and prostate cancer, and cognitive dysfunction (14). Isoflavones are among the major subgroups of phytoestrogens richly found in soybeans, germinated seeds, red grapes, soy flour, dates, cereals, and strawberries (15). Daidzein, a polyphenolic isoflavone, is one of the most important phytoestrogens with anti-oxidant, anti-inflammatory, and anti-fibrotic effects in the cisplatin (cis-diaminedichloroplatinum II, CDDP)‐induced kidney injury and bleomycin-induced pulmonary fibrosis (16, 17).

This study aimed to evaluate the effects of daidzein compared with E2 (as a positive control) on UUO-induced renal function, fibrosis, glomerulosclerosis, and apoptosis and its interaction with AT1 and Mas receptors in ovariectomized (OVX) rats as a model of postmenopausal women. Moreover, we assessed whether the expression of pro-fibrotic miR-33a and miR-27a and their downstream target the α1 chain of collagen type 3 (Col3A1) play a role in the interaction between daidzein and AT1 and Mas receptors. 

## Materials and Methods

***Drugs***

The AT1R antagonist losartan (sc-353662) and daidzein (sc-24001A) were purchased from Santa Cruz Biotechnology (Santa Cruz, CA, USA). A779 (MasR antagonist; SML1370) and dimethyl sulfoxide (DMSO, D2650) were purchased from Sigma-Aldrich (St. Louis, MO, USA). 17-β-estradiol was obtained from Abureyhan Pharmaceutical Company (Tehran, Iran). 

***Experimental groups and surgical procedures***

**Figure 1:**
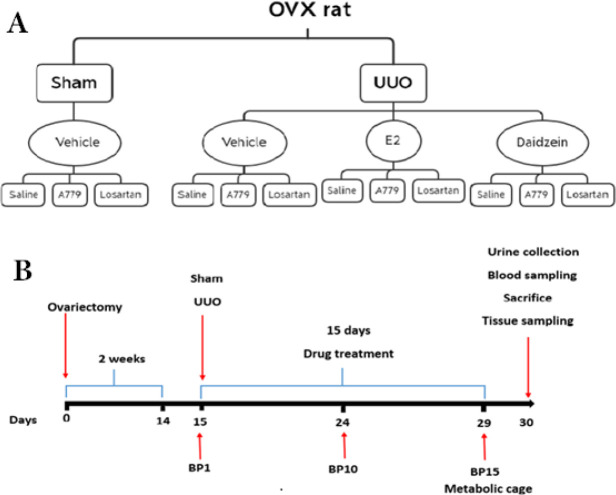
Schematic representation of the experimental groups of female rats included in the study (A). Timeline of the experimental protocol (B). BP: blood pressure measurement during treatment days UUO: Unilateral ureteral obstruction

The experimental protocol was carried out in compliance with the ARRIVE guidelines and was reviewed and approved by the ethical committee of the University of Kerman for animal welfare and studies (Ethic code: IR.KMU.REC.1397.249). Female rats (9 wk old, weight 180–220 gr) were obtained from the animal house of Kerman University of Medical Sciences (Kerman, Iran). The animals were housed in cages with free access to food and water under 24±2 ^°^C temperature and a 12 hr light/dark cycle. Two weeks before the main experiment (UUO), all animals underwent ovariectomy to produce similar conditions (e.g., estrogen and progesterone deficiency) as in postmenopausal women (18, 19). For ovariectomy, rats were anesthetized with ketamine/xylazine (80/10 mg/kg) intraperitoneally (IP), and a transverse incision (2 cm) was made in the lower abdominal region between the umbilicus and vagina. The abdomen’s skin and muscles were opened, and both ovaries were removed. In the end, 1-2 ml of physiological saline solution was poured into the abdomen, and muscles and skin were sutured (20). After two weeks, the OVX rats underwent UUO (21). Briefly, the animals were anesthetized with ketamine/xylazine (80/10 mg/kg, IP), and a left flank incision was made. The left ureter was exposed and ligated at two points 1 cm apart with 4-0 silk. Then the ureter was cut between the two ligatures to prevent urinary tract infection. In the sham-operated groups, all surgical procedures were the same as in the UUO groups but without ureteral ligation. The UUO animals were randomly divided into four main groups (n=21): sham+Vehicle (DMSO 1%), UUO+Vehicle, UUO+17β-Estradiol (E2) (5 μg/kg) (positive control group) (Dixon and Maric 2007), and UUO+daidzein 1 mg/kg. Each main group comprised three subgroups (n=7) of treatment with saline, A779 (744 μg/kg) (22) or losartan (10 mg/kg) (22) for 15 days. E2 was injected every four days to mimic the natural estrous cycle (23). All treatments were injected IP. Schematic representation of experimental groups and study timeline is illustrated in Figure 1A, B.

***Determination of optimum dose of daidzein***

**Figure 2:**
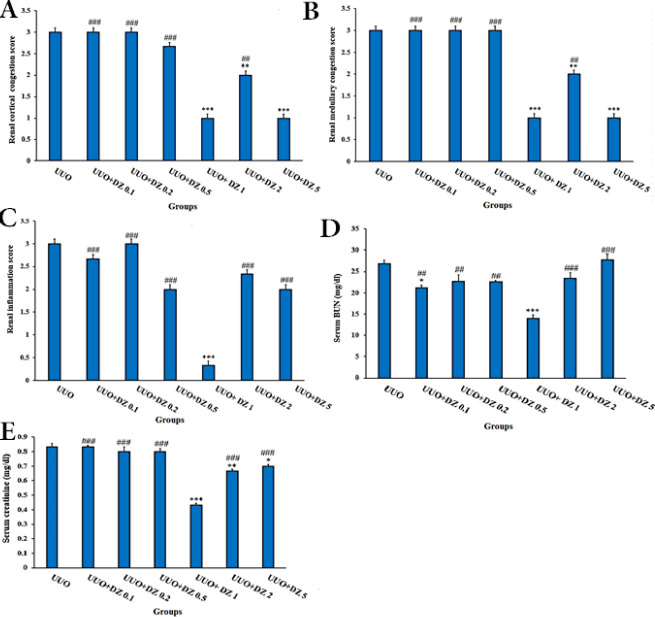
Pilot dose-response study to determine the optimal dose of daidzein for treatment of the ovariectomized UUO rats

The optimum dose of daidzein was determined with a pilot dose-response study. Thirty-five OVX-UUO rats were randomly split into seven groups (n=5) of UUO-vehicle and UUO-daidzein with doses of 0.1, 0.2, 0.5, 1, 2, and 5 mg/kg IP for 15 days. Finally, cortical and medullary renal congestion, renal inflammation, serum BUN (blood urea nitrogen), and serum creatinine were assessed (Figure 2A-E). The left/right kidney weight ratio significantly decreased in groups that received daidzein 1 mg/kg (data not shown). According to the results, a dose of 1 mg/kg of daidzin was selected as the optimum dose and was used for the rest of the study. The reason for the weaker effects of daidzein at doses higher than 1 mg/kg is not known at present and needs an independent investigation to explore.

***Blood pressure measurement and sample collection***

The systolic blood pressure (SBP) and diastolic blood pressure (DBP) were recorded with the tail-cuff method by the LE5002 non-invasive blood pressure system (Harvard Apparatus, Australia) on days 15, 24, and 29 of treatment. Before recording the pressure, animals were placed into a dark restrainer, and the pressure cuff was placed on the animal’s tail. The pressures were recorded five times from each animal at each time point and the average was calculated. On day 29, animals were placed in the metabolic cage for collecting the 24-hour urine sample. On day 30, animals were anesthetized by ketamine/xylazine (80/10 mg/kg, IP). The blood samples were collected from the left ventricle, centrifuged, and the serum was frozen at −20 °C. The rats were euthanized, and the kidneys were removed and weighed. The left/right kidney weight was measured. One-half of the left (UUO) kidney was fixed in 10% buffered formalin (pH 7.4) for histological assessment. The other half was frozen in liquid nitrogen and stored at -80 ^°^C for gene and protein expression assessment.

***Assessment of urine and serum parameters***

The urine and serum levels of creatinine were measured by standard kits (Pars Azmoon, Tehran, Iran) using an autoanalyzer (Selectra-XL, Vital Science, Netherlands). The urine and serum sodium, potassium, and calcium levels were measured by flame photometry (Corning, Halstead, Essex, UK). Fractional excretion of sodium (FE_Na+_), potassium (FE_K+_), and renal failure index (RFI) were calculated according to the following formulas (24).

FENa+=UNa inmmollit×PCr (inmgdl)PNa inmmollit×UCr (inmgdl)×100

RFI=UNa mmollit×PCr (mgdl)UCr (mgdl)×100

Where U and P mean urine and plasma respectively, and Cr means creatinine. For FE_K+_ the corresponding values for potassium were incorporated in the formula.

***Renal histology***

The left kidney tissues were dehydrated, embedded in paraffin, and cut into 5 μm-thick sections. Masson’s Trichrome staining was performed for collagen deposition assessment. Renal interstitial fibrosis index (RIF index) was scored from 0 to 3 as follows: 0=absent, 1=less than 25% of the area, 2=25–50% of the area, and 3=more than 50% of the area. From each rat, 20 fields at ×400 magnification (Olympus microscope, CX41, Tokyo, Japan) were visualized and scored by a blinded pathologist using Image J software (National Institutes of Health, Bethesda, MD, USA) (25). Periodic acid-Schiff (PAS) staining was used for scoring the degree of glomerulosclerosis. The scoring scales (0 to 4) were defined as follows: 0=no sclerosis of the glomeruli, 1=sclerosis up to 25%, 2=sclerosis 25% to 50%, 3= sclerosis 50% to 75%, and 4= sclerosis >75% (26). Twenty-five to fifty non-overlapping glomeruli were selected randomly for each animal at ×400 magnification.

***Immunohistochemistry (IHC)***

Four µm paraffin sections of the left kidneys were deparafﬁnized in xylene and rehydrated in graded alcohol and distilled water. Sections were kept in 3% H_2_O_2_ for 7 min to block endogenous peroxidase in a dark and humid condition and afterward washed with PBS. Primary cleaved caspase 3 antibody (1:150, RBK009-05, Zytomed, Germany) was incubated overnight at 4 ^°^C. Primary antibody binding was detected by incubation with HRP-labeled appropriate secondary antibody (Mouse/Rabbit UnoVue Detection System, Netherlands). After immersion in 3,3-diaminobenzidine (DAB), the sections were counterstained with hematoxylin. Finally, ten fields were selected randomly, and images were taken by an Olympus microscope (CX41, Tokyo, Japan) with ×400 magnification. The percentage of positive cells was scored by the pathologist in a blinded setting as: 1=<5%; 2= 5–25%; 3= 26-50%; and 4= 51-75% and the intensity of staining was scored as: 0= negative; 1=weak; 2=intermediate; and 3=severe. The final score (0-12) was obtained by multiplying the percent of positive cells by the intensity score (27).

***Quantitative real-time PCR (qRT-PCR)***

Left kidney tissues were homogenized by an ultrasonic homogenizer (UP 200H, Germany), and total RNA (including small RNA) was extracted using the RNeasy Mini Kit (Qiagen, Hilden, Germany) according to the manufacturer’s instructions. The RNA concentrations were measured by a NanoDrop ND-1000 spectrophotometer (Isogen Life Science, Utrecht, Netherlands). Total RNA was reverse-transcribed using the high-capacity cDNA reverse transcription kit (Applied Biosystems, Foster City, CA, USA). Then quantitative polymerase chain reactions (qPCR) were performed using predesigned prime time qPCR probe assays from Integrated DNA Technologies (IDT, Leuven, Belgium) (Table 1). RNA U6 (RNU6) and GAPDH were used as the internal control for miRNAs and genes, respectively. The fold change in expression was calculated using the comparative CT method (2^−ΔΔCT^) (28). 

***Western blot analysis***

The tissue of the obstructed kidneys was homogenized in ice-cold radio-immunoprecipitation assay (RIPA) buffer and incubated for 30 min. The samples were centrifuged at 12,000 x g, 4 ^°^C for 20 min, and their protein concentration was determined using the bicinchoninic acid (BCA) method. Proteins were separated by size using SDS-PAGE and then transferred onto a polyvinylidene fluoride (PVDF) membrane. The PVDF membrane was incubated in the blocker solution overnight at 4 ^°^C. In the next step, the PVDF membranes were incubated with anti-COL3A1 (GTX60940) and anti-glyceraldehyde 3-phosphate dehydrogenase (GAPDH) (ab181602) for 3 hr at room temperature. After being washed three times with Tris-buffered saline with 0.1% Tween 20 (TBST) (5 min each time), the PVDF membrane was incubated with horseradish peroxidase (HRP)-conjugated goat anti-mouse IgG (SC-516102) or anti-rabbit IgG (PZ5610) for 1 hr at room temperature. After washing with TBST, enhanced chemiluminescence (Thermo Fisher Scientific, Inc.) was used to observe the protein bands in a dark room. GAPDH was used as housekeeping control, and the protein band density was quantified using Image J software. 

***Statistical analysis***

The results are expressed as mean±SEM, and all statistical analyses were performed using SPSS v26.0 (IBM Corporation, Armonk, NY, USA). The data normality was checked with the Kolmogorov-Smirnov test. A three-way ANOVA statistical analysis with Tukey’s *post hoc* test was applied for normally-distributed data. For non-normally-distributed data, the Kruskal-Wallis test followed by the Mann-Whitney U test was used. For blood pressure analysis, three-way repeated-measures ANOVA was used to analyze the time, model (Sham, UUO), and treatment interactions**.** Mauchly’s test was used to assess sphericity, and non-sphericity was corrected using the Greenhouse-Geisser correction. Statistical comparisons over time were made by one‐way repeated measures ANOVA followed by Tukey’s *post hoc* test. *P*<0.05 was considered statistically significant.

## Results

***Daidzein inhibits the increase in kidney weight and renal failure index in UUO rats***

**Figure 3:**
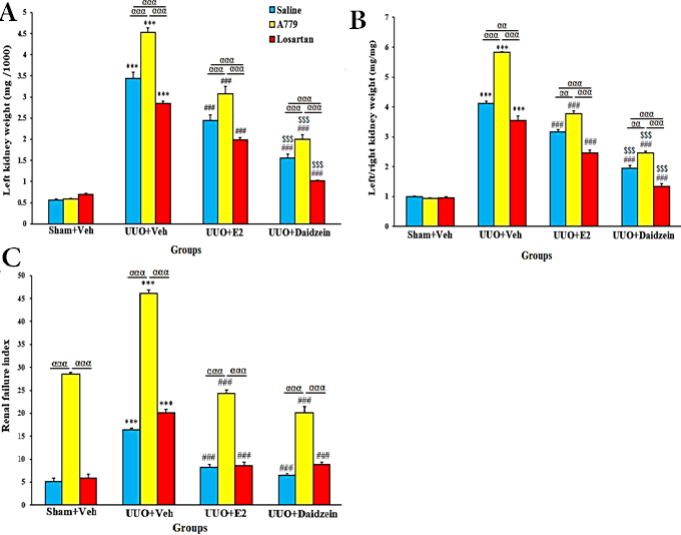
Effect of daidzein on the left kidney weight of ovariectomized UUO rats (A), left/right kidney weight ratio (B), and RFI (C)

The left kidney weight and left/right kidney weight ratio in UUO+Veh groups were significantly increased compared with Sham+Veh groups (*P*<0.001, Figure 3A, B) so that A779 further increased and losartan decreased these parameters (*P*<0.05). E2 (5 µg/kg) or daidzein (1 mg/kg) alone and E2 or daidzein in the presence of A779 or losartan reduced these variables (*P*<0.001), while daidzein was more effective than E2 (*P*<0.001). Daidzein in co-treatment with A779 prevented the A779 worsening effects, and in co-treatment with losartan further decreased these parameters (*P*<0.001). The RFI index also showed a significant increase in UUO+Veh groups (*P*<0.001) and treatment with E2 or daidzein alone or when co-administered with A779 or losartan significantly improved this index compared with UUO+Veh groups (*P*<0.001) (Figure 3C). A779 potentiated UUO-induced RFI in all groups (*P*<0.001).

***Daidzein reduces the systolic and diastolic blood pressure in UUO rats***

**Figure 4:**
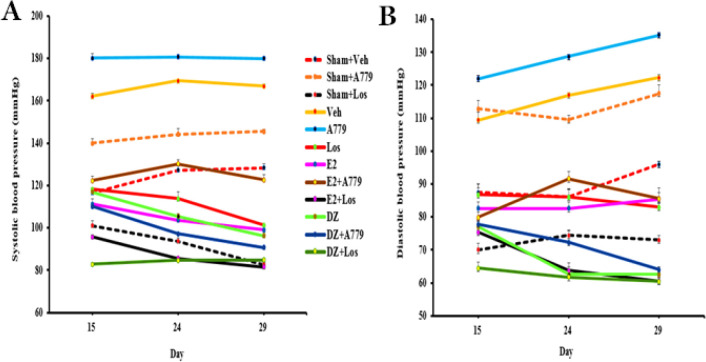
Effect of daidzein on SBP (A) and DBP (B) of ovariectomized UUO rats

The UUO procedure significantly increased SBP and DBP )at all-time points( compared with the related sham groups (*P*<0.001) (Figure 4A, B). Losartan treatment significantly decreased SBP and DBP in all groups, while A779 had the opposite effect (*P*<0.001). Also, treatment with E2 or daidzein with or without A779 or losartan reduced SBP and DBP of UUO rats (*P*<0.001) (Figure 4A, B). The UUO+A779 group showed the highest blood pressure, while the UUO+DZ+Los group had the lowest blood pressure.

***Effect of Daidzein treatment on renal interstitial fibrosis index and glomerulosclerosis***

**Figure 5:**
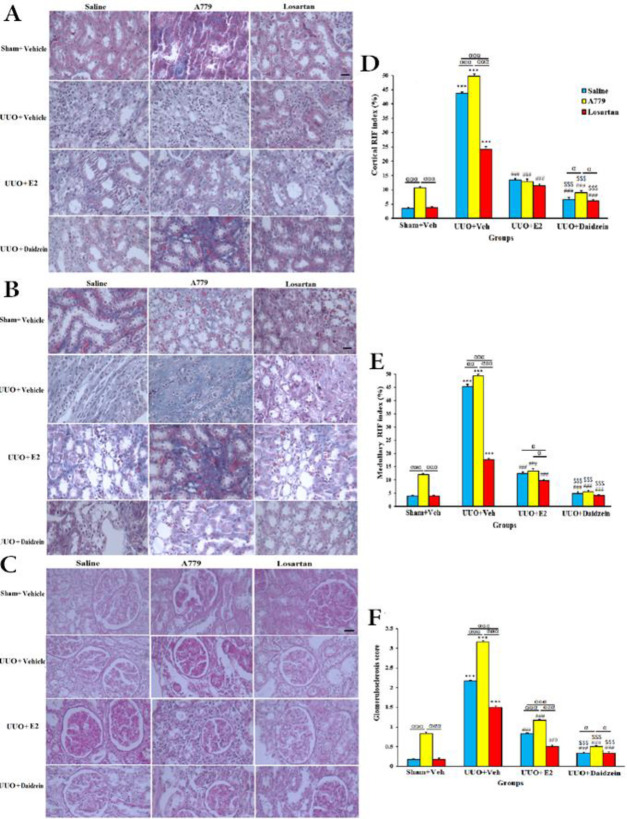
Representative Masson’s trichrome staining for renal collagen deposition in the cortex (A) and medulla (B) of left kidney in animal groups. Representative PAS-stained tissues for glomerulosclerosis (C). Quantification (mean±SEM) of renal fibrosis represented by the RIF index in the cortex (D) and medulla (E). Quantification of glomerulosclerosis (F). Original magnification X400; Scale bar: 100 um. (n=7 for each group)

In the sham+Veh groups, A779 increased cortical and medullary RIF index and glomerulosclerosis (*P*<0.001). These pathologic damages were increased significantly in the UUO+Veh groups compared with sham+Veh groups (*P*<0.001). A779 further increased and losartan decreased these parameters (*P*<0.05) (Figure 5A-F). E2 or daidzein alone or co-treatment with A779 or losartan markedly reduced cortical and medullary RIF index and glomerulosclerosis (*P*<0.001) (Figure 5A-F). Daidzein was also more effective than E2 in reducing these parameters (*P*<0.001). Daidzein in co-treatment with A779 prevented the A779 worsening effects, and in co-treatment with losartan further decreased these parameters (*P*<0.001).

***Daidzein inhibits renal cell apoptosis***

**Figure 6:**
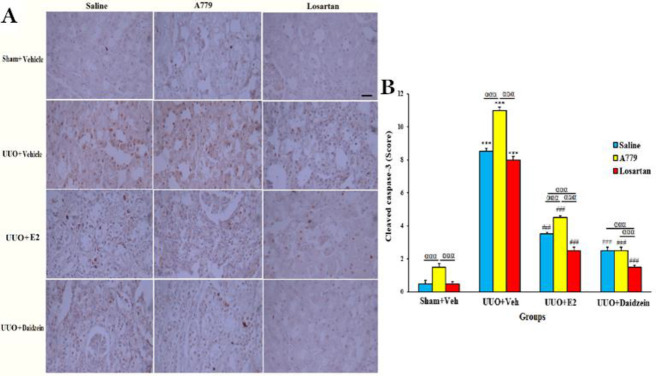
Effect of daidzein on cleaved caspase-3 in sham and UUO groups. Representative cleaved caspase-3 immunostaining (A). Quantification (mean ± SEM) of cleaved caspase-3 (B). Original magnification X400; Scale bar: 100 um. (n=7 for each group). ***P<0.001 vs Sham+Vehicle. ^##^*P<*0.01 and ^###^ *P<*0.001 vs UUO+Vehicle. ^ααα^*P<*0.001 significant difference between subgroups

The immunohistochemical assessment revealed that cleaved caspase-3 increases with A779 treatment in sham+Veh groups (*P*<0.001). In UUO+Veh groups cleaved caspase-3 significantly increased compared with sham+Veh groups with the highest level in A779 groups, indicative of enhanced renal cell apoptosis due to MasR blocking (*P*<0.001, Figure 6A, B). E2 or daidzein alone or in combination with A779 or losartan reduced the cleaved caspase-3 expression compared with the related UUO+Veh group (*P*<0.001). Co-treatment with A779 masked the augmenting fibrosis effects of A779, and co-treatment with losartan potentiated the ameliorative effect of daidzein (*P*<0.001).

***Daidzein reduces FE***_Na+_ ***, FE***_K+_ ***, and urine calcium levels in UUO rats***

**Figure 7:**
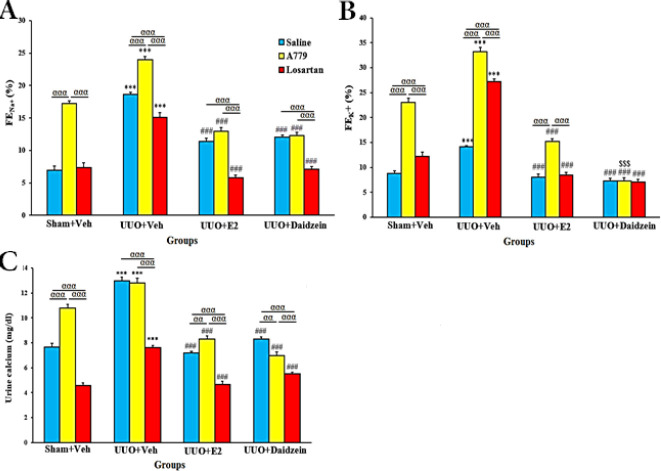
Effect of daidzein on FE_Na+_ (A), FE_K+_ (B), and urine calcium (C). Data are mean ± SEM, n=7. *** *P<*0.001 vs sham+Vehicle. ^###^*P<*0.001 vs UUO+Veh. ^$$$^ *P<*0.001 vs UUO+E2. ^αα^ *P<*0.01 and ^ααα^*P<*0.001, significant differences between subgroups

Figure 7A-C depicts the effect of daidzein and E2 on FE_Na+_, FE_K+_, and urine calcium in the study groups. In the sham+Veh groups, A779 increased FE_Na+_, FE_K+_, and urine calcium (*P*<0.001). Compared with sham+Veh groups, these parameters were increased significantly in UUO+Veh groups with the highest level by A779 treatment (*P*<0.001). E2 and daidzein alone or combined with A779 or losartan substantially reduced FE_Na+_, FE_K+_, and urine calcium levels (*P*<0.001). Daidzein+A779 reduced FE_K+_ more efficiently (*P*<0.001) than E2+A779 treatment.

***Daidzein treatment of UUO rats reduces the expression of miR-33a and miR-27a***

**Figure 8:**
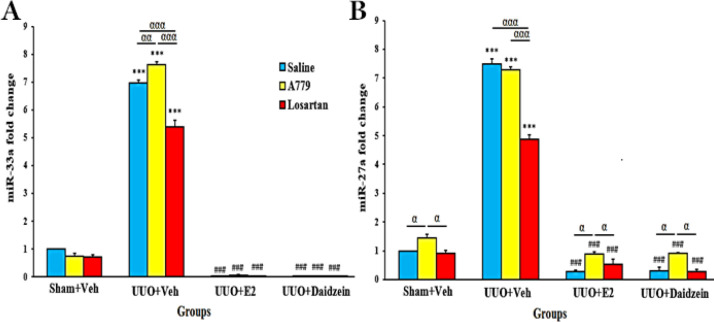
Relative expression of miR-33a (A) and miR-27a (B) in sham and obstructed kidneys of ovariectomized UUO rats. Mean±SEM (n=7)

In the sham+Veh groups, A779 increased miR-27a (*P*<0.05). The expression of miR-33a and miR-27a increased significantly in UUO rats (*P*<0.001). A779 increased miR-33a further and losartan decreased the expression of both miRs (*P*<0.05). Daidzein or E2 with or without A779/losartan significantly reduced the expression of miR-33a and miR-27 in UUO rats (*P*<0.001), even to levels lower than that in sham rats (Figure 8A, B). 

***Daidzein treatment of UUO rats resulted in the reduction of COL3A1 expression***

**Figure 9:**
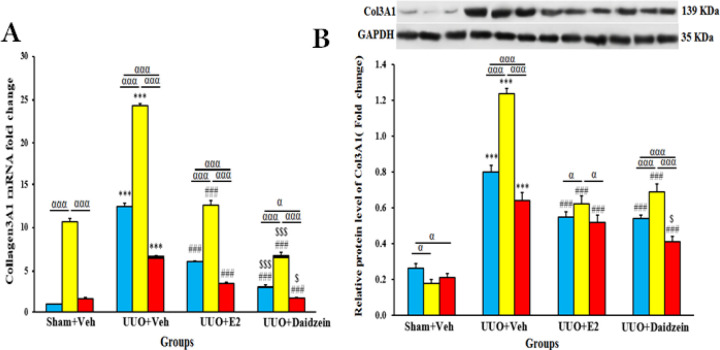
Relative expression of COL3A1 mRNA and protein (A, B) in sham and obstructed kidneys. Mean±SEM (n=7)

UUO maneuver increased the expression of COL3A1 mRNA and protein significantly. The expression of COL3A1 mRNA experienced another step of increase by A779 treatment in sham+Veh and all UUO groups (*P*<0.001). A779 also caused a further elevation in protein expression of COL3A1 in the UUO groups (*P*<0.001) (Figure 9A). These effects were attenuated by daidzein or E2 alone or with co-treatment with A779 or losartan (*P*<0.05). Notably, daidzein was more effective than E2 in recovering COL3A1 mRNA expression towards its normal level in the sham+Veh group (*P*<0.05). 

Renal cortical congestion score (A), renal medullary congestion score (B), renal inflammation (C), serum BUN (D), and serum creatinine (E). Data are mean±SEM (n = 5). *P<0.05, **P<0.01, ***P<0.001 compared with the UUO group. ^##^*P<*0.01, ^###^*P<*0.001 compared with the UUO+DZ1 group

UUO: Unilateral ureteral obstruction

**Table 1:** Primer sequences and probe assay identification (ID)

**Gene name Sequence and assay ID Reference **
miR-33a-F CGCGCGTGCATTGTAGTTG (29) miR-33a-R CACCAGGGTCCGAGGT miR-33a-RT 5′-TGGATATCCACACCAGGGTCCGAGGTATTCGGTGTGGATATCCATGCAATG
miR-27a-F 5' CGGCGGTTTCACAGTGGCTAAG 3' (30) miR-27a-R 5' CCAGTGCAGGGTCCGAGGTAT 3' miR-27a-RT 5' GTCGTATCCAGTGCAGGGTCCGAGGTATTCGCACTGGATACGACGCGGAA 3'
RNU6 F: 5′-CTCGCTTCGGCAGCACA-3’ (31) R: 5′-AACGCTTCACGAATTTGCGT-3’ RT: AACGCTTCACGAATTTGCGT
COL3A1 Rn.PT.58.11138874
GAPDH Rn.PT.39a.11180736.g

Data are mean ± SEM, n=7. *** *P<*0.001 vs Sham+Vehicle. ^###^ *P<*0.001 vs UUO+Vehicle. ^$$$^ *P<*0.001 vs UUO+E2. ^αα^ *P<*0.01, ^ααα^ *P<*0.001, significant differences between subgroups

RFI: Renal failure index; UUO: Unilateral ureteral obstruction, E2:17β-estradiol

Data are mean±SEM, n=7. All groups are ovariectomized. Sham (non-UUO) groups are in dotted lines, and UUO groups are in continuous lines

Veh: Vehicle; Los: Losartan; DZ: daidzein. SBP: Systolic blood pressure. DBP: Diastolic blood pressure; UUO: Unilateral ureteral obstruction

***P<0.001 vs corresponding Sham+Vehicle. ^#^*P<*0.05, ^##^*P<*0.01, and ^###^*P<*0.001 vs corresponding UUO+Vehicle. ^$$$^ *P<*0.001 vs the corresponding UUO+E2. ^α^*P<*0.05, ^αα^*P<*0.01, and ^ααα^*P<*0.001, significant differences between subgroups

UUO: Unilateral ureteral obstruction, E2:17β-estradiol

UUO: Unilateral ureteral obstruction, E2:17β-estradiol

UUO: Unilateral ureteral obstruction, E2:17β-estradiol

*** *P<*0.001 vs Sham+Vehicle. ^###^ *P<*0.001 vs UUO+Vehicle. ^α^ *P<*0.05, ^αα^ *P<*0.01, and ^ααα^ *P<*0.001, significant differences between subgroups

UUO: Unilateral ureteral obstruction; E2:17β-estradiol

*** *P<*0.001 vs Sham+Vehicle. ^###^ *P<*0.001 vs UUO+Vehicle. ^$^ *P<*0.05 and ^$$$^ *P<*0.001 vs UUO+E2. ^α^ *P<*0.05 and ^ααα^ *P<*0.001, significant difference between subgroups

COL3A1: Collagen3A1; UUO: Unilateral ureteral obstruction; E2:17β-estradiol

## Discussion

In the present study, we investigated the effect of daidzein and estrogen (E2) alone and in combination with A779 or losartan on UUO-induced renal fibrosis in OVX rats, a model of postmenopausal conditions in women. The main findings are that daidzein (or E2): 1- significantly decreased kidney congestion, FE_Na+_, FE_K+_, urine calcium, and systolic and diastolic blood pressure in UUO rats, 2- inhibited UUO-induced miR-33a and miR-27a overexpression, 3- ameliorated glomerulosclerosis and renal cell apoptosis, decreased the RIF index and RFI; and 4- inhibited Col3A1 mRNA and protein expression. 5- Daidzein (or E2) in co-treatment with A779 in UUO rats, masked the worsening effects of A779 on these parameters. 6- Co-treatment with losartan in UUO rats further decreased these parameters. 

Obstructive kidney disease is one of the major causes of renal interstitial fibrosis and glomerulosclerosis leading to CKD (32), due to the accumulation of major ECM components such as collagen-3 in the interstitium (2, 33). These processes mainly result from activation of the RAS axis and inactivation of Ang1-7 and are driven by tissue expression of TGF-β1(2, 7). Concordantly, we observed severe ECM deposition and glomerulosclerosis in obstructed kidney tissue, and daidzein (and E2) treatment alone or combined with A779 or losartan profoundly mitigated these effects. 

Regarding apoptosis, immunohistochemical staining of cleaved caspase-3 in obstructed kidneys showed severe renal cell apoptosis, and A779 treatment caused the most apoptotic effect. Daidzein (and E2) alone or combined with A779 decreased the expression of cleaved caspase-3 and when combined with losartan further decreased the expression of these parameters, indicative of reduced renal cell apoptosis via interaction with angiotensin AT1 and Mas receptors. Consistently, it has been shown that the RAS system causes apoptosis in renal tubular cells via AT1R in UUO nephropathy (34, 35). The anti-apoptotic effects of daidzein have been reported in experimental models of bleomycin-induced pulmonary fibrosis and cisplatin-induced nephrotoxicity (16, 17, 36). In the present study MasR blocking promoted renal weight gain, RIF, cell apoptosis, electrolyte urinary excretion, and mRNA expression of collagen3A1. Losartan mitigated these parameters, and daidzein in the presence of MasR blockade (A779) and AT1R blockade (losartan) alleviated all of these parameters, with losartan being more effective than A779. It seems that these effects of daidzein might be induced by a MasR-independent mechanism. In line with this hypothesis, it has been shown that the production of anti-inflammatory cytokines via CD4^+^ T-cells is stronger in the female gender (37), and pretreatment with daidzein improves renal injury via reduction in proinflammatory cytokines TNF-α and IL-6 in cisplatin-induced nephrotoxicity (17). Concordantly in our recent study on the UUO kidney model, daidzein significantly reduced oxidative stress and inflammation. Daidzein treatment was accompanied by an increase in anti-oxidants superoxide dismutase (SOD), glutathione peroxidase (GPx), and catalase activity, and a decrease in the oxidant malondialdehyde (MDA), and in inflammatory cytokines of tumor necrosis factor-alpha (TNF-α), IL-6, and IL-1β (38). The other possibility is that the more efficient the renal protective effect of daidzein and E2 masks the weaker the anticipated harmful effects of MasR blockade. More studies are needed to clarify this hypothesis. Mechanistically, E2 increases nitric oxide (NO) production via a G protein-coupled estrogen receptor (GPER) through phosphorylation of endothelial nitric oxide synthase (eNOS), involving ERK1/2 and PI3K/AKT signaling pathways (39). In this relationship cross-talk of estrogen receptors (ER) and MasR has been reported to involve in NO production (40). In diabetes Mellitus-induced myocardial fibrosis in OVX rats, apoptotic cells, and fibrosis were attenuated by a GPR30 agonist that was accompanied by reduction of cardiac iNOS activity and NO level (41). A renoprotective effect is proposed for estradiol GPCR1 activation via regulation of ECM production, renal hypertrophy, glomerular permeability, and Na^+^ excretion (42). It is possible that the effects of daidzein are mediated by this type of receptor and by similar mechanisms. Daidzein may also induce its protection against renal fibrosis via more recently introduced pro- and anti-inflammatory transcription regulating factors, namely microRNAs. It is known that the RAS receptors modulate miRNA production and are affected by miRNAs too (43). In line with this, we found that daidzein treatment down-regulated pro-fibrotic miR-33a expression and decreased the expression of its target gene collagen3. Among treatments, A779 and losartan were the most potent in increasing and decreasing miR-33 expression, respectively. MasR blockade also caused the largest increase in mRNA and protein expression of collagen3 but AT1 blockade decreased RNA and protein expression of collagen3. Daidzein treatment was so effective that it masked the worsening effect of A779. However, losartan further decreased the expression of collagen 3 already reduced by daidzein. Consistently in patients and rats with type 2 diabetic nephropathy, the miR-33 expression has been associated with renal inflammation and fibrosis via activating the NF-κB/TGF-β pathway (5, 44).

miR-27a is also a pro-fibrotic miRNA that plays a crucial role in kidney disease pathophysiology (45) and is involved in RAS activation, inflammation, oxidative stress, and apoptosis (46). In diabetic nephropathy, miR-27a was related to mitochondrial dysfunction and endoplasmic reticulum stress (47). Consistently, we observed a miR-27a overexpression in the obstructed kidneys, and daidzein (and E2) treatment inhibited its overexpression. Again, among treatment modalities, A779 had the largest effect on miR-27 expression while losartan reduced the expression of this miR effectively. 

## Conclusion

Overall, our results indicate that daidzein attenuates renal dysfunction, fibrosis, glomerulosclerosis, and apoptosis in the UUO model of OVX rats through a decrease in AT1R activation, an increase in MasR function, and inhibition of miR-33a and miR-27a overexpression. Daidzein is highly efficient, as even in the presence of MasR antagonist A779, it improved kidney function, and histology, i.e., it masked the anticipated harmful effects of MasR blockade. Co-treatment with losartan further improved the ameliorating effect of daidzein on kidney damage. Based on these observations, daidzein might be an interesting candidate for treating CKD in postmenopausal and older women, especially when co-treated with losartan. 

## Authors’ Contributions

HN provided supervision, conceptualization, methodology, funding acquisition, writing-reviewing and editing, and validation. SS provided conceptualization, methodology, writing-reviewing, and editing. MA helped with investigation, data curation, formal analysis, writing, and original draft preparation. SJF, SR, and EJ provided investigation. FP helped with funding acquisition, investigation, formal analysis, data curation, software, reviewing, and editing. SY, PP, and SS provided funding acquisition, reviewing and editing, and validation. All authors proofread and approved the final manuscript. The authors declare that all data were generated in-house and that no paper mill was used.

## Conflicts of Interest

The authors declare that no conflicts of interest exist.
